# Association of history of cerebrovascular disease with severity of COVID-19

**DOI:** 10.1007/s00415-020-10121-0

**Published:** 2020-08-06

**Authors:** Timo Siepmann, Annahita Sedghi, Jessica Barlinn, Katja de With, Lutz Mirow, Martin Wolz, Thomas Gruenewald, Sina Helbig, Percy Schroettner, Simon Winzer, Simone von Bonin, Haidar Moustafa, Lars-Peder Pallesen, Bernhard Rosengarten, Joerg Schubert, Andreas Gueldner, Peter Spieth, Thea Koch, Stefan Bornstein, Heinz Reichmann, Volker Puetz, Kristian Barlinn

**Affiliations:** 1 Department of Neurology, University Hospital Carl Gustav Carus, Technische Universität Dresden, Fetscherstraße 74, 01307 Dresden, Germany; 2 Division of Infectious Diseases, University Hospital Carl Gustav Carus, Technische Universität Dresden, Dresden, Germany; 3 Department of General and Visceral Surgery, Klinikum Chemnitz gGmbH, Chemnitz, Germany; 4 Department of Neurology, Elblandklinikum Meißen, Meißen, Germany; 5 Department of Infectious Diseases/Tropical Medicine, Klinikum Chemnitz gGmbH, Chemnitz, Germany; 6 Department of Virology, University Hospital Carl Gustav Carus, Technische Universität Dresden, Dresden, Germany; 7 Department of Internal Medicine III, University Hospital Carl Gustav Carus, Technische Universität Dresden, Dresden, Germany; 8 Department of Neurology, Klinikum Chemnitz gGmbH, Chemnitz, Germany; 9 Department of Hematology and Oncology, Elblandklinikum Riesa, Riesa, Germany; 10 Department of Anesthesiology and Intensive Care, University Hospital Carl Gustav Carus, Technische Universität Dresden, Dresden, Germany

**Keywords:** Cerebrovascular disease, Stroke, Prognosis, Critical care, COVID-19

## Abstract

**Objective:**

To determine whether a history of cerebrovascular disease (CVD) increases risk of severe coronavirus disease 2019 (COVID-19).

**Methods:**

In a retrospective multicenter study, we retrieved individual data from in-patients treated March 1 to April 15, 2020 from COVID-19 registries of three hospitals in Saxony, Germany. We also performed a systematic review and meta-analysis following PRISMA recommendations using PubMed, EMBASE, Cochrane Library databases and bibliographies of identified papers (last search on April 11, 2020) and pooled data with those deriving from our multicenter study. Of 3762 records identified, 11 eligible observational studies of laboratory-confirmed COVID-19 patients were included in quantitative data synthesis.

Risk ratios (RR) of severe COVID-19 according to history of CVD were pooled using DerSimonian and Laird random effects model. Between-study heterogeneity was assessed using Cochran’s *Q* and I2-statistics. Severity of COVID-19 according to definitions applied in included studies was the main outcome. Sensitivity analyses were conducted for clusters of studies with equal definitions of severity.

**Results:**

Pooled analysis included data from 1906 laboratory-confirmed COVID-19 patients (43.9% females, median age ranging from 39 to 76 years). Patients with previous CVD had higher risk of severe COVID-19 than those without [RR 2.07, 95% confidence interval (CI) 1.52–2.81; *p* < 0.0001]. This association was also observed in clusters of studies that defined severe manifestation of the disease by clinical parameters (RR 1.44, 95% CI 1.22–1.71; *p* < 0.0001), necessity of intensive care (RR 2.79, 95% CI 1.83–4.24; *p* < 0.0001) and in-hospital death (RR 2.18, 95% CI 1.75–2.7; *p* < 0.0001).

**Conclusion:**

A history of CVD might constitute an important risk factor of unfavorable clinical course of COVID-19  suggesting a need of tailored infection prevention and clinical management strategies for this population at risk.

**Electronic supplementary material:**

The online version of this article (10.1007/s00415-020-10121-0) contains supplementary material, which is available to authorized users.

## Introduction

Rapid transmission of the severe acute respiratory syndrome coronavirus 2 (SARS-CoV-2) and a case fatality rate that is up to 40 times higher than mortality of seasonal influenza make coronavirus disease 2019 a global threat [1, 2]. The latter is largely explained by high risk of acute respiratory distress syndrome as well as sepsis, multi-organ failure and disseminated intravascular coagulopathy, which is most pronounced in the elderly and in premorbid patients with a cardiovascular risk profile [3, 4]. In fact, patients with severe course of COVID-19 have up to threefold higher rates of pre-existing cardiovascular morbidity than patients with mild or moderate clinical manifestations [5].

Investigation of early cohorts of COVID-19 patients in China focused on the effects of classic cardiovascular risk factors such as arterial hypertension and coronary heart disease or comorbidity in general [6, 7]. By contrast, the importance of cerebrovascular disease (CVD) in the clinical course of COVID-19 is poorly understood. This is a relevant research gap as patients with CVD are particularly vulnerable toward pulmonary and inflammatory complications due to their frequent disability [8, 9]. At this stage of the pandemic, where overall comorbidity has been established as substantial risk factor, in-depth characterization of particularly endangered individuals might help design tailored infection prevention plans. Therefore, we aimed to assess whether history of CVD is associated with severe COVID-19. To approach this question, we assessed individual multicenter data from three cohorts of COVID-19 patients treated during the first months of the pandemic in Germany. In order to assess consistency among regions and increase generalizability of our findings, we then went on to pool our data with published data of COVID-19 patients who were being treated in Wuhan and other regions in China.

## Methods

### Multicenter study

#### Study design and subjects

**Fig. 1:**
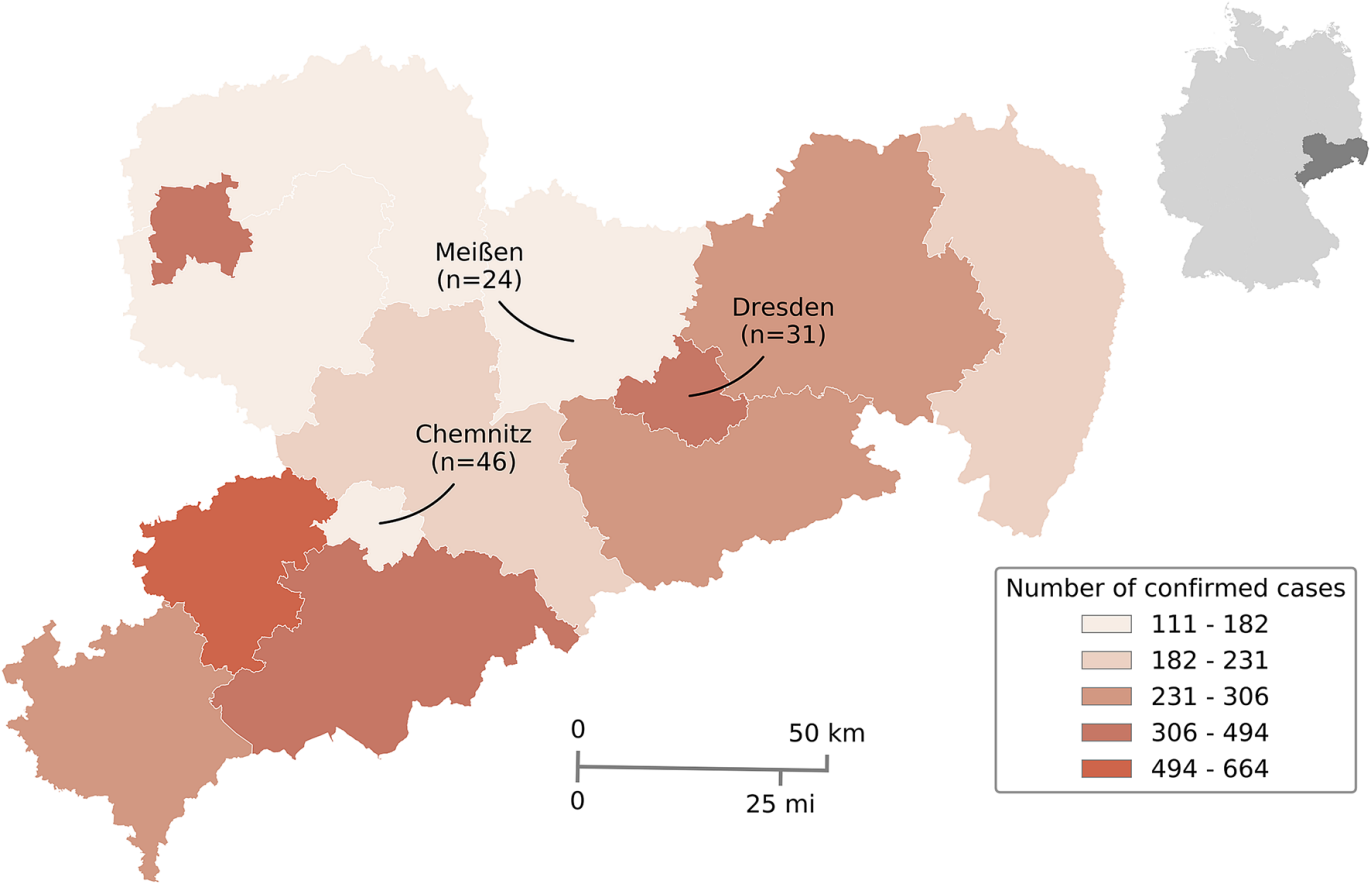
Map of study sites in Germany where individual data on patients with COVID-19 were retrieved. Location of participating sites in Saxony, Germany, with rates of confirmed infections with SARS CoV-2 based on epidemiological data provided by Robert Koch Institute as of April 15, 2020 (www.rki.de/EN/Home/homepage_node.html). Numbers in brackets refer to absolute numbers of patients included in the multicenter cohort

In a retrospective multicenter study, consecutive patients ≥ 18 years with laboratory-confirmed diagnosis of COVID-19 who have been admitted to the three participating hospitals (University Hospital Carl Gustav Carus Dresden, Klinikum Chemnitz gGmbH, Elblandklinikum Meißen) in Saxony, Germany between March 1 and April 15, 2020 were selected from the ongoing COVID-19 registries. Locations of participating hospitals are illustrated in Fig. 1. Laboratory tests for detection of SARS-CoV-2 included real-time reverse transcription polymerase chain reaction (RT-PCR) assays (RealStar® SARS-CoV-2 RT-PCR Kit RUO, Altona Diagnostics, Hamburg, Germany; Allplex™ 2019-nCoV Assay, Seegene Inc., Seoul, Republic of Korea; GeneFinderTM COVID-19 Plus RealAmp, Osang Healthcare Co., Gyeonggi-do, Republic of Korea) on respiratory specimen from nasal or oropharyngeal swab. We obtained data on age, sex and vascular comorbidities including arterial hypertension, hyperlipidemia, diabetes mellitus, atrial fibrillation, coronary heart disease, tobacco use and past history of CVD. Cerebrovascular disease was subdivided into ischemic stroke, transient ischemic attack and intracerebral hemorrhage. We also reviewed medical records and neuroimaging reports from cranial computed tomography or magnetic resonance imaging studies for evidence of previous clinically apparent or silent CVD. We detected one patient with evidence of previous lacunar stroke on cranial magnetic resonance imaging that was not diagnosed history of CVD and decided to include this patient in our analysis.

#### Severity outcomes

In order to assess the association of past history of CVD and risk of severe clinical course of COVID-19, we aimed to classify all patients in our multicenter cohort into “severe” and “non-severe” COVID-19. However, previously published approaches to categorize severity of COVID-19 were found to be inconsistent and all together three different most frequently reported approaches could be identified. In order to achieve comparability of outcome data with previously published cohorts, we separately applied these methods to dichotomize our patients into severe and non-severe clinical course of the disease. First, we classified severity of COVID-19 in the patients of our multicenter cohort based on clinical parameters according to the classification by the National Health Commission guidelines on the Diagnosis and Treatment of COVID-19 [10]. In this classification, “mild” was defined as mild clinical symptoms with no signs of pneumonia on chest imaging; “moderate” as fever, respiratory symptoms with radiologic signs of pneumonia; “severe” as respiratory distress with respiratory rate ≥ 30 per minute and/or oxygen saturation at rest ≤ 93% and/or oxygenation index ≤ 300 mmHg and/or progression of pulmonary lesion size > 50% within 48 h, “critical” as respiratory failure requiring mechanical ventilation, hemodynamic shock, or any other organ failure with necessity of intensive care.

We also categorized stages of disease by using the Lean European Open Survey on SARS-CoV-2 Infected Patients (LEOSS) definition, comprising the following disease stages: “uncomplicated”, asymptomatic or symptoms of upper respiratory tract infection, nausea, emesis, diarrhea, fever; “complicated”, need for oxygen supplementation, partial arterial oxygen pressure at room air < 70 mmHg, oxygen saturation at room air < 90%, aspartate aminotransferase or alanine aminotransferase > 5-fold upper limit normal, new cardiac arrhythmias, new pericardial effusion > 1 cm, new heart failure with pulmonary edema, congestive hepatopathy or peripheral edema; “critical”, need for catecholamines, life-threatening cardiac arrhythmia, invasive or non-invasive mechanical ventilation, liver failure with < 50% Quick value (equaling approximately > 1.55 international normalized ratio), quick Sequential [Sepsis-Related] Organ Failure-Assessment score ≥ 2, renal failure in need of dialysis; “recovery”, improvement by one phase and defervescence [11]. Second, severity of COVID-19 was also dichotomized for in-hospital death versus survival with death equaling severe and survival indicating non-severe course. Third, patients were classified into severe or non-severe clinical course of COVID-19 based on whether they required intensive care unit (ICU) treatment or underwent regular in-patient care until discharge.

### Literature search and study eligibility

This systematic review and meta-analysis complied with the Preferred Reporting Items for Systematic Reviews and Meta-Analyses (PRISMA) recommendations [12]. We systematically searched electronic databases including MEDLINE (accessed by PubMed), EMBASE and Cochrane Library for identification of all available observational studies that reported on laboratory-confirmed COVID-19 patients aged ≥18 years with information given on disease severity and past history of CVD. In addition, bibliographies of identified full-text articles and those of relevant review articles were searched manually. In order to be exhaustive, we limited our search on electronic databases to search term “COVID-19” with combinations of associated medical subject headings (MeSH) “COVID-2019”, “severe acute respiratory syndrome coronavirus 2”, “2019-nCoV”, “SARS-CoV-2”, “2019nCoV”, “Wuhan”, “coronavirus”, “2019/12”. The complete search algorithm is provided in Online Resource 1.

Our systematic search covered publications from the earliest date available until our last search date April 11, 2020. No language or other restrictions were imposed. All identified articles were screened using the following eligibility criteria: (1) observational cohorts consisting of a minimum of five patients ≥ 18 years who have been hospitalized for COVID-19 laboratory-confirmed by nasal or oropharyngeal swab RT-PCR; (2) data available on past history of CVD; (3) categorization of COVID-19 severity according to study-specific outcome definitions.

Assessment of identified articles involved three steps: screening of titles, abstracts and full texts by two independent reviewers (T.S. and K.B.). Any disagreements were resolved by consensus. Abstracts that did not provide sufficient information for analysis of methodology were subject to full-text evaluation. In case of missing information or any obscurities, the corresponding authors of the identified articles were contacted for clarification.

Two reviewers (T.S. and K.B.) independently extracted data on included studies from the full-text articles with insertion into a standardized data extraction form (Excel, Microsoft, Redmond, WA, USA). Extracted variables were first author, publication year, study design, sample size, demographic values, vascular comorbidities including history of CVD as well as definition of severity outcomes of COVID-19 and respective absolute numbers of outcome events.

### Rating of the quality of evidence

We used the Oxford Centre for Evidence-based Medicine Rating Scale to assess the quality of evidence in the included individual studies [13]. Quality assessment was independently performed by two investigators (T.S. and K.B.) and disagreements were resolved by consensus.

### Statistical analysis

#### Multicenter study

In the multicenter study*,* continuous and non-continuous variables are presented as median with interquartile range (IQR) for skewed data and percentages for proportional data. Between-group comparisons were performed using Chi-square test, Fisher's exact test and Mann–Whitney *U* test, where applicable. Multivariable logistic regression was performed to explore the predictive value of history of CVD for severity outcomes of COVID-19 including clinical severity according to the classification by the National Health Commission guidelines on the Diagnosis and Treatment of COVID-19, in-hospital death and necessity of intensive care [10]. Candidate variables were identified from the between-group comparisons, whereas a *p* value of ≤ 0.25 was used for covariate inclusion in the multivariable model. The final model was conducted using a backward selection procedure, whereas covariates with a *p* value <0.1 were removed from the model.

#### Pooled analysis

In the quantitative data synthesis, risk ratios (RR) and their corresponding 95% confidence intervals (95% CI) for history of CVD were calculated from the absolute numbers of patients with severe and non-severe COVID-19 outcomes as provided by each study. In our main analysis, we used a composite dichotomized outcome of severity subsuming all severity outcomes that were reported by each of the included studies comprising severity based on clinical parameters, in-hospital death versus survival and necessity of intensive care versus regular in-patient care. If included studies classified severity outcome based on clinical parameters into more than two categories (e.g., mild, moderate, severe, critical), those were subsumed under severe (i.e., severe and critical) and non-severe (i.e., mild and moderate) categories. Thus, in our main analysis, all patients reported in studies identified from literature search cohort were classified into severe or non-severe COVID-19 based on the classification used by each study. With respect to our multicenter study, we chose to apply the approach of defining severity by clinical parameters as recommended by the National Health Commission guidelines on the Diagnosis and Treatment of COVID-19 since this tended to be the most widely acknowledged method in the literature [10]. Continuity correction of 0.5 was used for studies with a zero cell [14]. If a study reported two or more zero-cell events, it was excluded from respective analysis. DerSimonian and Laird random effects model was used to compute the pooled RR for included studies. [15]

In order to allow separate assessment of clusters of studies with equal definitions of COVID-19 in conjunction with our multicenter data, sensitivity analyses were conducted for severity outcomes. We clustered studies that used the same approach to define severity and pooled these data with our multicenter data by applying the same severity definition to our local cohorts. Analyses were carried out for three clusters of studies: first, studies defining severity based on clinical parameters; second, studies defining severity based on necessity of intensive care; third, those defining severity by in-hospital death.

Assuming that only available cases with complete data on disease severity outcomes were reported in included studies, pairwise deletion method was used to handle missing outcome data. Between-study heterogeneity was assessed using Cochran’s *Q* test and *I*^2^ statistics, where *I*^2^ values of 0–40% indicated absent or low, 30–60% moderate, 50–90% substantial and 75–100% considerable heterogeneity [16]. Significance level of heterogeneity was set at *p* < 0.1. Publication bias was assessed by visual inspection of funnel plot and Egger's linear regression test. Statistical significance was set at *p* < 0.05. All statistical analyses were conducted using STATA software package (Version 12.1, StataCorp., College Station, TX).

## Results

### Multicenter study

During the observational period from March 1 to April 15, 2020, 101 patients (48.5% females, median age 66 [55–78]) with laboratory-confirmed COVID-19 have been admitted to participating hospitals. Two patients were still hospitalized at the time of data analysis. Overall, 74 of 101 (73.3%) patients showed severe or critical clinical course with necessity of ICU treatment in 23 of 101 (22.8%) patients and in-hospital death in 20 of 99 (20.2%) patients. In the entire multicenter cohort, a history of CVD was evident in 16 (15.8%) patients with higher frequencies in patients with severe course of COVID-19 when applying the National Health Commission guidelines on the Diagnosis and Treatment of COVID-19 with dichotomized severity categories subsuming categories mild, moderate in “non-severe” and categories severe and critical into “severe”. (20.3% vs. 3.7%, *p* = 0.06) [10]. A history of CVD was also found to be more frequent in patients with severe COVID-19 when severity was defined by necessity of intensive care vs. regular in-patient care (30.4% vs. 11.5%, *p* = 0.047) and in-hospital death vs. survival (35% vs. 11.4%, *p* = 0.02). A detailed description of demographic values, comorbidities and outcomes is shown in Table 1.

**Table 1:** Baseline demographic values, comorbidities and outcomes of multicenter cohort with COVID-19

(A) Baseline characteristics	COVID-19 (*n* = 101)	Severe (*n* = 74)	Non-severe (*n* = 27)	*p* value^a^
Demographic values				
Age, median (IQR)	66 (55–78)	72 (58–80)	53 (43–63)	< 0.001
Women, *n* (%)	49 (48.5)	31 (41.9)	18 (66.7)	0.04
Past vascular risk factors, *n* (%)				
Arterial hypertension	61 (60.4)	53 (71.6)	8 (29.6)	< 0.001
Hyperlipidemia	27 (26.7)	21 (28.4)	6 (22.2)	0.38
Diabetes mellitus	27 (26.7)	24 (32.4)	3 (11.1)	0.04
Atrial fibrillation	18 (17.8)	15 (20.3)	3 (11.1)	0.39
Tobacco use	13 (12.9)	7 (9.5)	6 (22.2)	0.1
Coronary heart disease	14 (13.9)	11 (14.9)	3 (11.1)	0.75
Cerebrovascular disease	16 (15.8)	15 (20.3)	1 (3.7)	0.06
Ischemic stroke	13 (12.9)	12 (16.2)	1 (3.7)	
Transient ischemic attack	2 (2)	2 (2.7)	0	
Intracerebral hemorrhage	1 (1)	1 (1.4)	0	

(B) Severity outcomes	COVID-19 (*n* = 101)			
Disease severity by NHC, *n* (%)				
Mild/moderate	27 (26.7)			
Severe	48 (47.5)			
Critical	26 (25.8)			
Stages of disease by LEOSS, *n* (%)				
Uncomplicated	28 (27.7)			
Complicated	46 (45.6)			
Critical	27 (26.7)			
Recovery	70 (69.3)			
Intensive care treatment	23 (22.8)			
In-hospital death	20/99 (20.2)^b^			

(A) The upper part of the table shows the distribution of demographic and vascular risk profiles among patients with severe versus non-severe COVID-19 as defined by the National Health Commission guideline with subsuming categories mild and moderate in a “non-severe” category and moderate and critical in a “severe “ category [10]

(B) The lower part of the table shows the distribution of severity outcomes within our cohort of COVID-19 patients

*IQR* interquartile range, *NHC* National Health Commission, *LEOSS* Lean European Open Survey on SARS CoV II Infected Patients

^a^*p* values refer to between-group comparisons

^b^According to patients discharged at the time of analysis

In multivariable analysis adjusting for selected covariates (i.e., age, sex, arterial hypertension and diabetes mellitus), past history of CVD emerged as an independent predictor of severity of COVID-19 when severity was defined by necessity of ICU treatment (adjusted RR 4.81; 95% CI 1.34-17.3; *p* = 0.02), but not by clinical severity (*p* = 0.55) or in-hospital death (*p* = 0.16).

### Systematic review

**Fig. 2:**
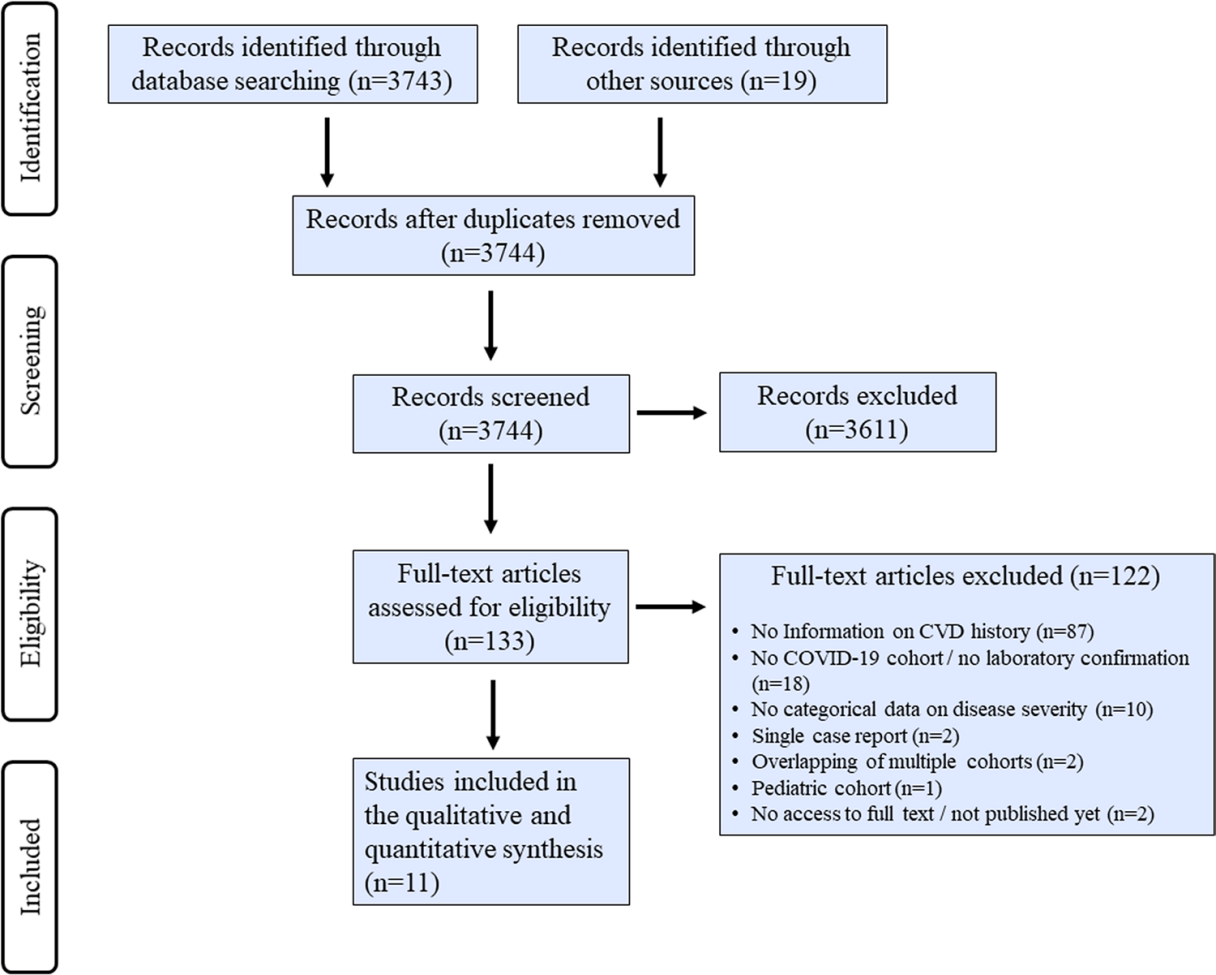
Flowchart on identification of studies on COVID-19 eligible for quantitative data synthesis. PRISMA flowchart illustrating systematic screening and selection process of published observational studies reporting on laboratory-confirmed COVID-19 patients with data available on disease severity and past history of CVD

A total of 3743 abstracts were retrieved from electronic databases and 19 from bibliographies of published literature. After exclusion of duplicates and articles that did not fulfill eligibility criteria, 11 studies comprising 1805 laboratory-confirmed COVID-19 patients (43.7% females, median ages ranging from 39 to 76 years) were included in quantitative data synthesis as described in detail in Table 2 [17–27]. The flowchart showing systematic screening and selection process is depicted in Figure 2. All studies included patients from China and were of descriptive observational design. In order to avoid overlapping patient populations, we excluded two multicentric reports on Chinese cohorts from our quantitative synthesis because they partially comprised data from the same hospitals that were reported by other studies included in our analyses [1, 28]. None of the included studies reported by what criteria history of CVD was defined. Seven studies reported severity outcomes based on in-hospital death (*n* = 5) [17, 18, 22–25] or necessity of intensive care (*n* = 2) [20, 21], whereas three studies [19, 26, 27] categorized clinical course of COVID-19 into “severe” and “non-severe” based on the National Health Commission guidelines on the Diagnosis and Treatment of COVID-19 [10]. One study defined severity by length of hospitalization with a cut-off of 10 days [22]. Overall distribution of demographic data and vascular risk profiles among these studies was highly congruent with data from our local German multicenter cohort with relatively high frequencies of pre-existing vascular risk factors, high ranges of median ages and a rather balanced male-to-female ratio.

**Table 2:** Study characteristics of included published studies

Study	Study design/quality	Severity outcomes	Study size, n	Median age (IQR), y	Female, %	History of CVD, %	History of diabetes, %	History of hypertension, %	Observational period
Cao et al., 2020^17, b^	Descriptive/4	Death vs. survival	17 vs. 85	72(18) vs. 53(19)	24 vs. 53	17.6 vs. 3.5	35.3 vs. 5.9	64.7 vs. 20.0	01/03–02/01/20
Chen et al., 2020^18^	Descriptive/4	Death vs. survival	113 vs. 161	68(15) vs. 51(29)	27 vs. 45	4.0 vs. 0.0	na	48 vs. 24	01/13–02/12/20
Feng et al., 2020^19, c^	Descriptive/4	Critical vs. severe vs. moderate	352 vs. 54 vs. 70	51(26) vs. 58(19) vs. 61(19)	46 vs. 39 vs. 31	11.4 vs. 1.9 vs. 2.3	21.1 vs. 9.1	49.6 vs. 20.7	01/01–02/15/20
Lei et al., 2020^20, d^	Descriptive/4	ICU vs. non-ICU	15 vs. 19	55(30) vs. 47(29)	67 vs. 53	13.3 vs. 0.0	40.0 vs. 10.5	60 vs. 21.1	01/01–02/05/20
Wang D et al., 2020^21, b^	Descriptive/4	ICU vs. Non-ICU	36 vs. 102	66(21) vs. 51(25)	39 vs. 48	16.7 vs. 1.0	22.2 vs. 5.9	58.3 vs. 21.6	01/01–01/28/20
Wang L et al., 2020^22,d^	Descriptive/4	Death vs. survival	65 vs. 274	76(17) vs. 68(10)	40 vs. 54	15.6 vs. 4.0	17.2 vs. 15.8	50.0 vs. 38.8	01/01–02/06/20
Xu et al., 2020^23^	Descriptive/4	Severe vs. non-severe	33 vs. 29	45(17) vs. 39(19)	42 vs. 45	3.0 vs. 0.0	3.0 vs. 0.0	12.0 vs. 3.0	01/10–01/26/20
Yang et al., 2020^24, c^	Descriptive/4	Death vs. survival	32 vs. 20	64.6(11.2) vs. 51.9(12.9)^e^	34 vs. 30	22 vs. 0.0	22 vs. 10	na	12/24/19–01/26/20
Yuan et al., 2020^25^	Descriptive/4	Death vs. survival	10 vs. 17	68(10) vs. 55(25)	60 vs. 53	10 vs. 0.0	60 vs. 0	50 vs. 0	01/01–01/25/20
Zhang et al., 2020^26^	Descriptive/4	Severe vs. non-severe	58 vs. 82	64(62) vs. 52(52)^f^	43 vs. 54	3.4 vs. 1.2	13.8 vs. 11.0	37.9 vs. 24.4	01/16–02/03/20
Zheng et al., 2020^27^	Descriptive/4	Severe vs. non-severe	30 vs. 131	57(20) vs. 40(20)	53 vs. 50	3.3 vs. 2.3	6.7 cs. 3.8	40 vs. 7.6	01/17–02/07/20

*TIA* transient ischemic attack, *ICU* intensive care unit, *IQR* interquartile range

^a^According to the quality rating scheme by the Oxford Centre for Evidence-based Medicine

^b^Overlapping cohort (publications on studies conducted at the same site introducing risk of overlap between study populations)

^c,d^Overlapping cohorts

^e^Mean ± standard deviation

^f^Range

Characteristics of included studies are detailed in Table 2.

### Quantitative data synthesis

**Fig. 3:**
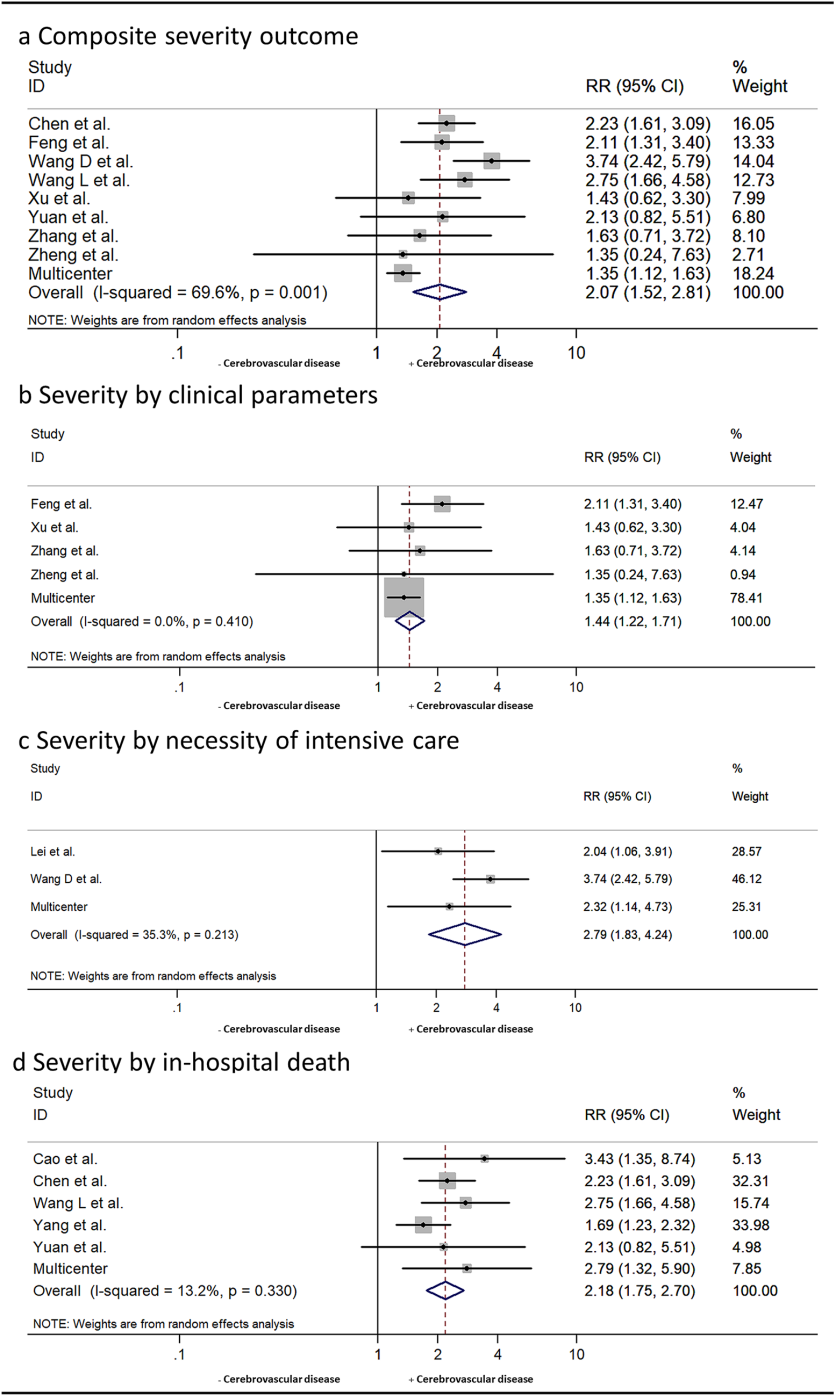
Association of history of cerebrovascular disease and severe clinical manifestation of COVID-19 among included studies. Forest plots illustrating associations of history of CVD and severe clinical manifestation of COVID-19 for composite severity outcome subsuming all definitions of severity as reported by included studies (**a**) as well as for clusters of studies defining severity by grading of clinical parameters (**b**), whether patients required intensive care (**c**), and in-hospital death (**d**). Composite outcome analysis as well as assessment of each cluster included only studies that have not shown any overlap in study populations during full text evaluation. Individual patient data from German multicenter cohort were evaluated for severity based on the Chinese Clinical Guidance for COVID-19 Pneumonia Diagnosis and Treatment

Pooled analysis including individual patient data from our multicenter cohort consisted of 1906 laboratory-confirmed COVID-19 patients (43.9% females, median age ranging from 39 to 76 years). Patients with a history of CVD had higher risk of severe COVID-19 than those without (RR 2.07, 95% CI 1.52–2.81; *p* < 0.0001) when using a composite dichotomized outcome of severity subsuming all severity outcomes that were reported by each of the included studies. We noted substantial heterogeneity between studies (*I*^2^=69%, *p* = 0.001, Figure 3). Consistently, an increased risk of severe COVID-19 in patients with a past medical history of CVD was also observed in sensitivity analyses separately considering clusters of studies that defined severity by clinical parameters (RR 1.44, 95% CI 1.22–1.71; *p < *0.0001), necessity of intensive care (RR 2.79, 95% CI 1.83-4.24; *p < *0.0001) and in-hospital death (RR 2.18, 95% CI 1.75-2.7; *p < *0.0001). Of these, evidence of low heterogeneity was observed for the ICU/non-ICU cluster (*I*^2^=35.3%, *p* = 0.21), whereas no heterogeneity was noted for the clinical parameters cluster (*I*^2^=0%, *p* = 0.41) nor the in-hospital death cluster (*I*^2^=13.2%, *p* = 0.33).

When considering only published data from Chinese cohorts in pooled analysis (*n* = 1805), history of CVD was also associated with increased risk of severity of COVID-19 (RR 2.39, 95% CI 1.94–2.94; *p < *0.0001) with similar results on sensitivity analyses for study-specific severity outcomes (clinical parameters: RR 1.83, 95% CI 1.28–2.63; *p* = 0.001; necessity of intensive care: RR 2.9, 95% CI 1.61-5.24; *p < *0.0001 and in-hospital death: RR 2.14, 95% CI 1.7–2.7; *p < *0.0001). While there was evidence of moderate between-study heterogeneity for additional analyses of the ICU/non-ICU cluster (*I*^2^=57.2%, *p* = 0.13), only low or absent heterogeneity was observed for the composite (*I*^2^=8.4%; *p* = 0.37), clinical severity (*I*^2^=0%; *p* = 0.83) and in-hospital death (*I*^2^=17.5%; *p* = 0.3) outcome clusters.

### Study quality

**Fig. 4:**
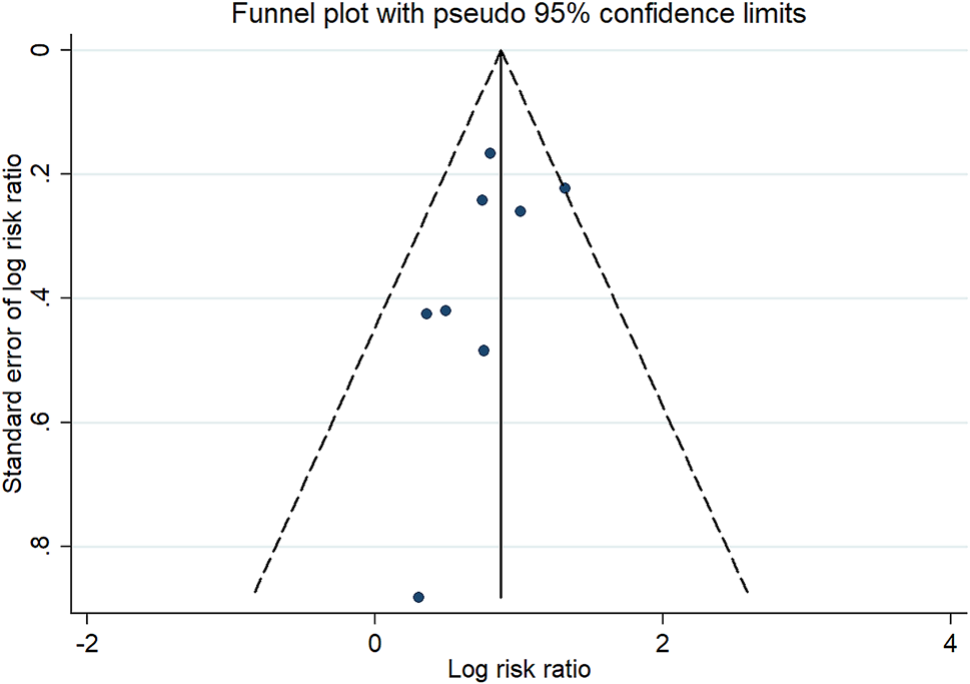
Assessment of publication bias. Visual inspection of funnel plot is not indicative of publication bias

According to Oxford Centre for Evidence-based Medicine Rating Scale Quality, all included studies from published literature were consistently graded as level of evidence 4. Visual inspection of funnel plot showed symmetry in both studies plotted near the average, and those more distant from the average depending on their precision, thus shaping a distribution which is not suggestive of publication bias (Figure 4). No small study effect was seen on Egger’s linear regression test (*p* = 0.26).

## Discussion

The major finding of our multicenter study is that a history of CVD is associated with an increased risk of developing severe course of COVID-19. This observation was consistent among pooled data which included descriptive observational studies from China during the rise of the pandemic and individual multicenter patient data from the first few months after the local outbreak in Germany.

A strength of our analysis is that we synthesized data from two countries both hit heavily by the pandemic and both showing consistent findings in our analyses with respect to association of history of CVD and measures of severity of COVID-19. Furthermore, data on distribution of demographic values as well as additional vascular comorbidities and their association with severity of COVID-19 was highly congruent between the German multicenter cohort and Chinese cohorts [17–27]. In particular, patients with severe COVID-19 were older, more frequently male and more often have had a history of arterial hypertension or diabetes. Consistency of our observations is also reflected by low evidence of heterogeneity among studies with equal severity outcomes indicating probable generalizability to other populations. Another strength of our synthesized analysis is the strict exclusion of overlapping study populations, which is especially important during the early phase of the pandemic where multiple descriptive studies were simultaneously derived from the ground zero region of Hubei, China. While an urgent need for data on the COVID-19 pandemic is apparent, it is important to reduce potential sources of bias that might skew pooled effect estimates [29].

Our study is limited by variance in definitions used for severity of COVID-19 among studies extracted from the literature. However, risk factor association for history of CVD found in pooled analysis using a composite severity outcome subsuming all study-specific outcomes was consistent with those deriving from individual sensitivity analysis of clusters of studies that applied the same definition of severity. Furthermore, data synthesized from the literature was limited by lack of explanation of how CVD was defined and therefore could not be analyzed regarding different types of cerebrovascular pathology in the context of COVID-19 prognosis. In our multicenter cohort, the majority of previous cerebrovascular accidents that led to being classified as history of CVD were ischemic and only one of 101 patients had previous intracerebral hemorrhage. Whether etiology of previous CVD relates to risk of COVID-19 severity requires further investigation. Data on pre-existing pulmonary disease were not available to an extent that would have allowed a separate analysis on how this might have influenced the observed association between a history of CVD and severity of COVID-19.

Lastly, the association of past history of stroke and COVID-19 severity was dependent on cardiovascular risk profile on multivariable analysis in our multicenter cohort when severity was classified using clinical parameters or in-hospital death versus survival. However, severity defined as necessity of intensive care showed an independent association with history of stroke.

This might be explained by differences in disease progression at the time of admission due to pulmonary vulnerability of stroke survivors [8, 9]. However, it needs to be acknowledged that we were not able to perform a multivariate analysis in the cohorts identified through literature research as individual patient data were not available. Therefore, the actual number of patients who have had an actual history of CVD included in our multicenter cohort was relatively small (*n* = 16). Moreover, individual descriptions of standardized critical care admission approaches among hospitals providing data to our multicenter analyses and those included in studies extracted from the literature were not consistently available. Therefore, a possible independency of the link between past history of CVD and COVID-19 severity requires further investigation. This analysis should be undertaken in cohorts with individual patient data available, preferably in the setting of a prospective observational study.

Since the outbreak of the pandemic, the impact of comorbidities on prognosis of COVID-19 has been extensively discussed with cardiovascular pathologies in the spotlight [5]. In particular, recent research has focused on traditional cardiovascular risk factors such as arterial hypertension and diabetes mellitus as predictors of disease severity [6, 7, 30]. However, it might be important to take a closer look into pre-existing brain vascular pathology of COVID-19 patients for several reasons. First, CVD is the leading cause of long-term acquired disability which increases the risk of pulmonary complications such as pneumonia [8, 9, 31]. While this association is not specific for infection caused by SARS-CoV-2, it might partly explain why in our analysis patients with a history of CVD displayed a higher risk of severe course of COVID-19, which is considered a primarily respiratory disease [32]. Second, accumulative evidence suggests that SARS-CoV-2 targets the central nervous system and may manifest with various neurological symptoms that might either be caused by direct neural damage or by neurovascular accident such as acute ischemic stroke [33]. From a pathophysiological perspective, SARS-CoV-2 appears to increase risk of cardiovascular events, possibly mediated by systemic inflammation compromising functional and structural integrity of the vasculature by inflammatory injury of the endothelium and increasing blood coagulability [34]. In patients, who already had experienced a cerebrovascular accident, brain vasculature might be at increased vulnerability toward these mechanisms. Lastly, patients with a history of CVD frequently have cardiovascular comorbidities that in turn might worsen prognosis of patients suffering from COVID-19 [28, 35].

Identification of populations at risk is one of the key factors in containing spread and reducing health care burden in epidemics [36]. This is even more important in a pandemic like COVID-19 where neither effective antiviral treatment nor vaccine is yet available to allow broad or targeted immunization of individuals at risk. In this context, knowing that a history of CVD increases risk of developing more severe disease manifestation upon infection with SARS-CoV-2, viewed in conjunction with previous data on comorbidity-related risk factor associations might be useful in designing risk-adapted prevention strategies.

Individuals who have a history of CVD are more likely to develop severe manifestation of COVID-19. Consistency among results in our pooled analyses indicates that this observation is generalizable beyond the studied regions in China and Germany. However, it remains to be answered whether the increased risk of severity observed in COVID-19 patients included in our analyses can be explained by a past history of CVD per se or simply reflects the additive effects of concomitant cardiovascular risk factors.

## Availability of data and material

Anonymized data will be shared by request from any qualified investigator.

## Electronic supplementary material

Below is the link to the electronic supplementary material.

Supplementary file1 (PDF 288 kb)
