# Supplementary material for: Association of history of cerebrovascular disease with severity of COVID-19
Source: J Neurol. 2020 Aug 6;268(3):773–84. doi: 10.1007/s00415-020-10121-0 (PMC7407424; doi:10.1007/s00415-020-10121-0)
Supplement: Supplementary file 1 — Supplementary file1 (PDF 288 kb) [file 415_2020_10121_MOESM1_ESM.pdf]

Online Resource: Complete search strings

Article title: Association of History of Cerebrovascular Disease with Severity of COVID-19

Journal: Journal of Neurology

Authors: Timo Siepmann, MD; Annahita Sedghi, MD; Jessica Barlinn, MD; Katja De With, MD, PhD; Lutz Mirow, MD; Martin Wolz, MD; Thomas Gruenewald, MD; Sina Helbig, MD; Percy Schroettner, MD; Simon Winzer, MD; Simone von Bonin, MD; Haidar Moustafa, MD; Lars-Peder Pallesen, MD; Bernhard Rosengarten, MD; Joerg Schubert, MD; Andreas Gueldner, MD; Peter Spieth, MD, MSc; Thea Koch, MD; Stefan Bornstein, MD; Heinz Reichmann, MD, PhD; Volker Puetz, MD; Kristian Barlinn, MD, MSc

Corresponding Author
Timo Siepmann, MD
Department of Neurology
University Hospital Carl Gustav Carus
Technische Universität Dresden
Fetscherstraße 74, 01307 Dresden
Germany
Phone: 0049-458-18578
timo.siepmann@ukdd.de

Search strings

Search strings applied to databases included the term „COVID-19" in combination with the following medical subject headings.

PubMED search string

„COVID-19" [All Fields] OR „COVID-2019" [All Fields] OR „severe acute respiratory syndrome coronavirus" [Supplementary Concept] OR „severe acute respiratory syndrome coronavirus 2" [All Fields] OR „2019-nCoV [All Fields] OR „SARS-CoV-2" [All Fields] OR „2019nCoV" [All Fields] OR ((„Wuhan" [All Fields] AND („coronavirus" [MeSH Terms] OR „coronavirus" [All Fields])) AND („2019/12"[PDAT] OR 2020[PDAT]))

EMBASE search string

„COVID-19" OR „COVID-2019" OR „severe acute respiratory syndrome coronavirus" OR „severe acute respiratory syndrome coronavirus 2" OR „2019-nCoV OR „SARS-CoV-2" OR „2019nCoV" OR „Wuhan" AND „coronavirus" OR „coronavirus" AND „2019/12" OR 2020

Cochran library search string

„COVID-19"
